# Prevalence, Severity of Extension, and Risk Factors of Gingivitis in a 3-Month Pregnant Population: A Multicenter Cross-Sectional Study

**DOI:** 10.3390/jcm12093349

**Published:** 2023-05-08

**Authors:** Jocelyne Gare, Aida Kanoute, Giovanna Orsini, Lucio Souza Gonçalves, Fahad Ali Alshehri, Denis Bourgeois, Florence Carrouel

**Affiliations:** 1 Health, Systemic, Process (P2S), Research Unit UR 4129, University Claude Bernard Lyon 1, University of Lyon, 69008 Lyon, France; jvgare@yahoo.fr (J.G.); denis.bourgeois@univ-lyon1.fr (D.B.); 2 Public Health Laboratory (LASAP), University Joseph Ki Zerbo, Ouagadougou 7021, Burkina Faso; 3 Public Health Service, Department of Dentistry, Cheikh Anta Diop University, Dakar 10700, Senegal; aida.kanoute@gmail.com; 4 Department of Clinical Sciences and Stomatology, Università Politecnica delle Marche, 60126 Ancona, Italy; g.orsini@staff.univpm.it; 5 Postgraduate Program in Dentistry, Estácio de Sá University, Rio de Janeiro 22790-710, Brazil; luciogoncalves@yahoo.com.br; 6 Department of Periodontics and Community Dentistry, College of Dentistry, King Saud University, Riyadh 12372, Saudi Arabia

**Keywords:** periodontal disease, gingivitis, pregnancy, microbiome, inflammation, dysbiosis, lifestyle, nutrition

## Abstract

The scope of this study was to assess the prevalence, severity of extension, and risk factors of gingivitis among pregnant women. In this cross-sectional study, 220 nulliparous women at 3 months of pregnancy were recruited in 2022 at the first obstetrical visit in Dakar, Senegal. Demographic characteristics, lifestyle habits, prenatal clinical status, and oral clinical parameters were recorded. Multivariable logistic regression modeling was used to assess relationships between gingivitis and risk factors. Eighty-eight percent of women had gingivitis, 15% were classified as moderate and 73% as severe. A total of 66.7% (95% CI [28.8–92.1]) of the sites had bleeding on interdental brushing. The odds for gingivitis decreased significantly for women consuming more than five portions of fruits and vegetables per day (OR = 0.15; 95% CI [0.03–0.66]) and increased in women who had a professional activity (OR = 6.75; 95% CI [1.27–35.87]) and high education. Concomitantly, the percentage of dental plaque (OR = 131.6; 95% CI [10.80–1619.71] and the severity of clinical attachment loss (OR = 7.70; 95% CI [3.16–18.92]) were important risk factors. Inverse associations were observed with increasing body mass index (OR = 0.76; 95% CI [0.63–0.93]). Our results underline that gingivitis cases and bleeding were particularly high among 3-month pregnant women. Literacy and adequate oral hygiene actions to modify behaviors and to achieve meticulous biofilm disorganization could make a favorable change in the gingival health outcome. Additionally, further research is necessary to precisely determine the role of biofilm-induced gingivitis and systemic-induced gingivitis in improving gingival conditions.

## 1. Introduction

Manifestations of a systemic condition are a source of nondental plaque biofilm-induced gingival diseases and usually do not resolve following plaque removal [1]. Contemporary literature emphasizes the multifactorial nature of the pathogenesis of developing periodontitis in pregnant women, for which the mechanism of origin is mainly related to the hormonal, genetic, and behavioral changes that are essential for normal fetal development [2]. These changes alter the mother’s microbiome in various body locations, including the gut, vagina, placenta, and oral cavity [3].

The oral microbiome during pregnancy appears influenced by oral and systemic conditions [4]. Complex biological reactions during pregnancy within the gingival tissues result from such increased levels of progesterone and estradiol hormones [5], action of local irritants, modification of eating, and the lack of proper hygiene measures, which are of great importance in the development of gingivitis and inflammation of the periodontal area [6,7]. The systemic risk or modifying factors affect the immune response to bacterial biofilm and drive vascular and gingival changes that may contribute to heightened gingival inflammation resulting in exaggerated or “hyper” inflammation in response to relatively small levels of oral biofilm [8]. Although the exact etiology is not fully understood, even without changes in the amount of oral biofilm present, the inflammation of the periodontal tissues due to the dysbiosis of the biofilm increases dramatically in severity during the course of a normal pregnancy [9].

Gingivitis, an early stage of periodontal disease, is the most common oral disease affecting 40–70% of patients in the gestational period [6,10,11,12,13,14]. Gingivitis occurred 1.81–2.2 times more frequently in pregnant women than in nonpregnant women [15,16]. Moreover, it is suggested in pregnancy that gingivitis does not predispose or progress to periodontitis [17].

Gingivitis generally appears between the 3rd and 8th months of gestation. In oral health, the scientific basis for evaluating pregnant women at 3 months is based on the physiological changes that occur in the mouth. In pregnancy, progesterone and estrogen drastically affect the periodontal process. As pregnancy progresses, the placenta begins to produce progesterone and estrogen. Early in pregnancy (first trimester), both estrogen and progesterone are overproduced by the corpus luteum [18]. Importantly, an increase in capillary permeability as a result of high estrogen levels in the blood predisposes pregnant women to gingivitis and hyperplasia [19]. Changes accompanying pregnancy in the first trimester frequently increase the body’s response to local inflammatory agents. Gingivitis is characterized by red, dark, swollen gingiva that bleeds easily and is, in fact, a sign of damaged vascularization [20].

If bacteria is necessary for initiating disease development, progression to gingivitis may be influenced by host environmental factors [21]. Studying the interrelatedness of gingival conditions, pregnancy, and their behavioral, physical, sociodemographic, biological, and oral health risk factors is important because the results can be used in developing interventions and policies to improve oral and general health [22].

The new classification of periodontal diseases and conditions aims to take a “precision medicine” approach and allows oral healthcare professionals to incorporate risk factors and their management [23]. A PubMed search with the terms “oral health AND pregnancy outcomes” returned 3.114 results, 92% after 2000 [24]. Although some research suggests a high incidence of periodontitis in pregnant women, the distribution and clustering of risk factors for gingivitis among pregnant women have not yet been adequately explored [11,25,26,27].

The scope of this study, aligning with the 2018 classification scheme, was to assess the prevalence, severity of extension and major risk factors of gingival conditions to the current understanding of pregnant women.

## 2. Materials and Methods

### 2.1. Study Design and Setting

This analysis was a part of the randomized controlled trial OP-PE protocol published by Kanoute et al. (2021) [28] and registered at ClinicalTrials.gov (NCT04989075). Two hundred and twenty women at 3 months of pregnancy were recruited between March 2022 and August 2022 at the first obstetrical visit to 6 National Hospital Centers of Dakar (Senegal).

This study was designed as a cross-sectional study. This research was performed in accordance with the STROBE guidelines (Supplementary Table S1). The study protocol was approved by the Review Committee of Dakar (Senegal) (protocol 000086/MSAS/CNERS/SP approved on 8 June 2021) and performed in accordance with the principles of the Declaration of Helsinki. Written informed consent was obtained from all participants.

### 2.2. Participants

Inclusion criteria were (i) pregnant women, (ii) between 18 and 35 years of age, (iii) from sub-Saharan Africa, (iv) nulliparous at the time of the obstetrical visit, (v) up to 12 weeks pregnant, (vi) acceptance of the terms and conditions of the study, and (viii) signature of the informed consent form.

The obstetric exclusion criteria were pregnant women (i) with uterine and vaginal congenital anomalies, (ii) who had premature termination of pregnancy for medical reasons, (iii) with fetal distress, and (iv) with infectious or systemic diseases, such as tuberculosis, HIV, cancers, candidiasis, and hematological diseases.

Oral exclusion criteria were pregnant women (i) with less than 20 natural teeth, excluding third molars, (ii) having none of the 4 premolar–molar pairs, (iii) with a history or treatment of PD, (iv) undergoing dental or orthodontic treatment, (v) with generalized (>30% of sites) stage II, III, and IV periodontal lesions (PD ≥ 4 mm, and/or CAL ≥ 4 mm), (vi) taking medications affecting the gingiva and/or oral mucosa, (vii) regularly using dental floss and/or interdental brushes and/or mouthwash, or (viii) unable to answer questions or noncooperative.

### 2.3. Outcomes

#### 2.3.1. Primary Outcome Measures

The primary outcome variable was to evaluate the prevalence and severity of extension of gingival inflammation cases in pregnant women.

#### 2.3.2. Secondary Outcome Measures

The secondary outcomes were to analyze the relationships between demographics, lifestyle factors, oral hygiene habits, oral clinical health status, and gingivitis cases among 3-month pregnant women.

### 2.4. Procedures

Participants were screened at their first prenatal visit. The study was proposed for pregnant women who met the inclusion criteria. In case of an agreement to participate and consent, the inclusion visit was planned at 3 months of pregnancy.

During the inclusion visit, participants signed an informed consent form, completed a questionnaire, and underwent obstetrical and oral clinical examinations. An electronic medical record (e-CRF Voozalyon 1.3; Voozanoo, Caluire, France) permitted us to record all information.

#### 2.4.1. Determination of Demographic and Behavioral Characteristics

Questionnaire assessments were used to obtain information on sociodemographic characteristics, lifestyle factors, oral hygiene habits, and medication use. Participants who declared eating less than five portions of fruits and vegetables per day, so at least 400 g (or 5 servings) of fruits and vegetables per day, were considered to have insufficient fruit and vegetable intake [29,30]. Participants who participated in less than the equivalent of 150 min of moderate-intensity (600 metabolic equivalents of task (MET)) physical activity per week were categorized as having insufficient physical activity. Sedentary is defined as inactive below 150 min of moderate weekly physical activity (i.e., 30 min per day, 5 days per week) or 75 min of vigorous physical activity (25 min, 3 days per week) [31].

#### 2.4.2. Determination of the Prenatal Clinical Status

The obstetric clinical examination included the measurement of height (cm) and weight (kg) using a calibrated clinical scale and stadiometer. The body mass index (BMI) was calculated and used to classify women as underweight (BMI < 18.5 kg/m^2^), healthy (18.5 kg/m^2^ ≤ BMI < 25 kg/m^2^), overweight (25.0 kg/m^2^ ≤ BMI < 30 kg/m^2^), and obese (BMI ≥ 30.0 kg/m^2^) [32].

#### 2.4.3. Determination of the Oral Clinical Parameters

Gingivitis on an intact periodontium and gingivitis on a reduced periodontium in a patient without a history of periodontitis are defined as ≥10% bleeding sites with probing depths ≤3 mm. Localized gingivitis is defined as 10–30% bleeding sites, and generalized gingivitis is defined as >30% bleeding sites [33].

Full-mouth clinical examinations, including probing depth (PD), clinical attachment level (CAL), gingival index (GI), and plaque index (PI), were carried out by a practitioner in a clinical center using a sterile U.S. Williams PDT sensor probe at a pressure of 20 g (Zila-Pro-Dentec Inc., Batesville, AR, USA) positioned parallel to the long axis of the tooth [34].

The bleeding on the interdental brushing index (BOIB) was recorded, as the bleeding response to the horizontal pressure applied in the interdental area by a calibrated interdental brush (IDB). After 30 s, bleeding at each gingival unit was recorded according to the following scale: 0, absence of bleeding after 30 s and 1, bleeding after 30 s [35,36]. The procedures for collecting bleeding data with a calibrated interdental brush are detailed by Bourgeois et al. [37].

All examiners with graduate training in periodontics were trained beforehand by at least one specialized periodontist in the use of the IAP Curaprox colorimetric probe (Curaden) and had obtained a minimum kappa value of 0.82 compared with the gold-standard examiner (excellent agreement according to the Landis and Koch scale) [38]. Examiners were blinded to each other, and two observations were collected at an interval of at least 15 min. The kappa statistic for the reproducibility of the pressure was 0.80. Intraexaminer reliability in using the dental-examination criteria was tested using the Kappa statistic. A 95% agreement on criteria for pocket depth and CAL was obtained. A visual analog scale (VAS) was used to estimate the correlation between the patient’s discomfort perception and the interproximal pressure (0 = no pain, 10 = unbearable pain) [39]. The pressure used to place the IDB was approximately 0.20–0.40 *g*-force, and 78% of participants were assigned a VAS score of ≤1.

Sound and carious teeth were recorded to calculate the DMFT (decayed, missing, and filled teeth) score for permanent teeth and identified caries-free women.

### 2.5. Statistical Analysis

Descriptive bivariate analyses between participant characteristics and the outcome, gingivitis, were evaluated using t-tests and logistic regression for continuous and binary/categorical variables, respectively. An exploratory analysis to check the behavior of the P-risk logit in relation to the variables age and BMI to discuss the need to introduce them in quantitative multivariate analysis was performed. Subsequently, an unconditional logistic model was run to screen the possible influencing factors from four broad domains: maternal characteristics, oral hygiene behaviors, oral clinical conditions, and socioeconomic characteristics. Only variables that generated a *p* value ≤ 0.20 in the unadjusted analyses were considered for the model, and they were retained in the multivariate logistic regression model. Considering the influence of age, professional activity, sedentary behavior, caries status, and daily frequency of tooth brushing on the results, they were also included as confounding factors in the multivariate analysis. Adjusted odds ratios (aORs) and 95% confidence intervals (CIs) were calculated. Multicollinearity analysis among independent variables was tested using a reference tolerance (TOL) < 0.1 and a variance inflation factor (VIF) > 5. All statistical tests were performed at a two-sided significance level of 0.05. The Shapiro–Wilk test was used to check the normality of the distribution (*p* value < 0.05). Statistical analyses were performed using XLSTAT 2022 software (Addinsoft, Paris, France).

## 3. Results

### 3.1. Demographic Characteristics and Clinical Parameters of Pregnant Women

**Figure 1:**
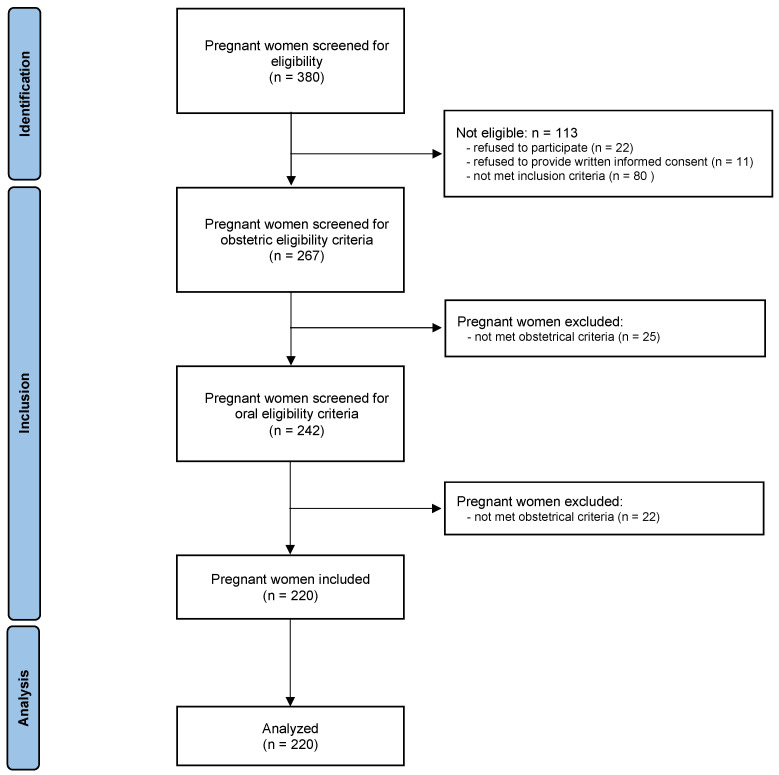
Flow chart of the study.

The study flow chart is presented in Figure 1. Of the 380 eligible pregnant women, 113 were ineligible, 25 did not meet obstetrical inclusion criteria, and 22 did not meet oral inclusion criteria. Thus, 220 pregnant women were included in the study.

The baseline characteristics of the pregnant women are presented in Table 1. Of the 220 women enrolled, 95% (n = 209) lived in an urban city. A total of 21.4% (n = 27) stated that they had never been to school. Their median age was 23 years (IQR 20–26), and most (63.2%, n = 139) had no professional activity. No women were current smokers, and 0.9% (n = 2) declared harmful alcohol use. The median BMI was 22.6 kg/m^2^ (IQR 22.2–25.1). Among pregnant women, 10.6% (n = 23) were classified as underweight, 63.3% (n = 140) normal, and 26.1% (n = 57) obese. During the first 3 months of pregnancy, 95.0% (n = 207) had sedentary behavior. A total of 98.9% of participants reported cleaning their teeth at least once with toothbrushes, with 84.3% (n = 183) reporting using toothbrushes alone, 1.4% (n = 3) using tooth sticks, and 14.6% (n = 31) using both. Almost 2/3 of pregnant women (74.5%, n = 161) declared that they consumed fewer than five fruits and vegetables per day.

### 3.2. Clinical Parameters of Pregnant Women

Table 2 details the clinical variables of the study population. A total of 68.9% of women had sound teeth, i.e., no cavities, no fillings, and no missing teeth due to cavities. All women (92.1%, n = 220) had a very low (<5) DMFT index. A total of 60.3% (n = 132) of women had at least one decayed tooth, and 35.2% (n = 77) had one or more missing teeth. The percentage of conservative care received was very low (6.4%, n = 14). A total of 93.0% of the population had at least one bleeding event with a median of 66.7% (IQR 28.8–92.1).

### 3.3. Prevalence of Gingivitis Cases in Pregnant Women

Pregnant women had gingivitis (gingival bleeding score ≥ 10%) in 88.2%, localized gingivitis in 15.0%, and generalized gingivitis in 73.2%. While 19% of the women had 100% of the sites bleeding, 7% had no bleeding on interdental brushing.

**Figure 2:**
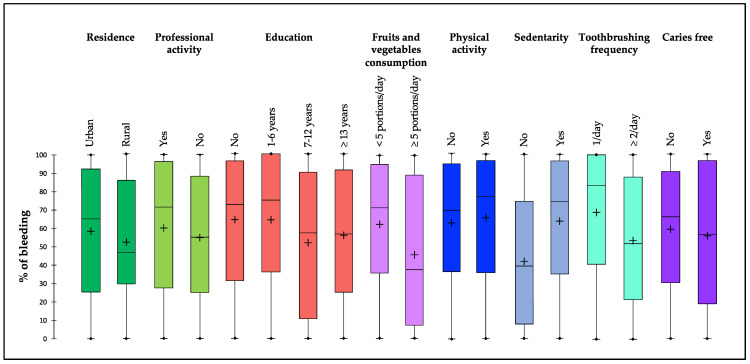
Percentage of bleeding on probing according to demographic characteristics, lifestyle habits, and clinical characteristics of pregnant women. Each box represents the first quartile, median quartile, and third quartile, from bottom to top.

Figure 2 describes the percentage of bleeding according to demographic characteristics, lifestyle habits, and clinical characteristics of pregnant women. Rurality, lack of professional activity, and education of more than 7 years were associated with a lower median percentage of BOIB. Eating five or more portions of fruits and vegetables per day, not being sedentary, and brushing teeth more than twice a day were lifestyle habits associated with a lower median percentage of bleeding. The absence of caries was a clinical feature associated with a lower median percentage of bleeding.

**Figure 3:**
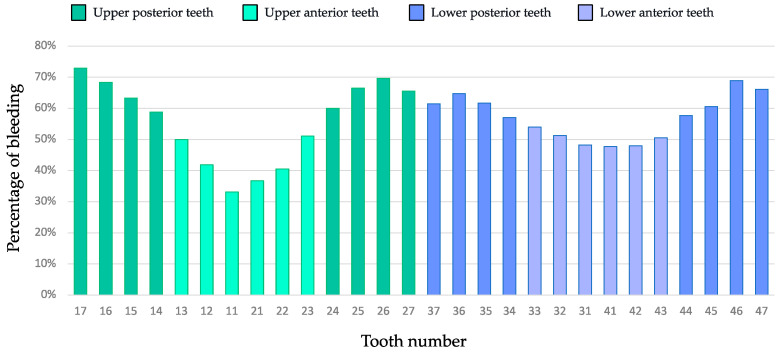
Percentage of pregnant women with bleeding per tooth. Each histogram bar represents a tooth. The green bars correspond to the top tooth, and the purple bars correspond to the bottom tooth.

The percentage of bleeding by tooth type is described in Figure 3. Among the 5413 sites registered, all patients combined, 56.4% (N = 3040) of sites showed bleeding. The highest bleeding scores were mostly observed in molars from the upper and lower arches. The upper right second molar was the tooth with the highest prevalence (72.9%), while the upper right incisor had the lowest prevalence (33.2%). The distribution of the bleeding prevalence by teeth in the mandible appears to be more homogeneous, mainly due to the higher occurrence of incisor–canine teeth compared to the upper group.

### 3.4. Distribution of No/Mild or Localized or Generalized Gingivitis Cases and Sociodemographic, Behavioral, and Clinical Variables

The correlation analysis between no/mild, localized, and generalized gingivitis is presented in Table 3. BMI was the only variable that had a significant association with gingival inflammation severity (*p* < 0.03). The prevalence of moderate–severe gingivitis in women with 1–6 years of education was 24.2% (8/33) and 14.4% (23/161), respectively, while moderate–severe gingivitis was observed in 33% (11/33) and 38.8% (62/161) of women with more than 12 years of education, respectively. There were no statistically significant differences in the prevalence of gingivitis between the urban and rural populations (*p* = 0.457) or between women with professional activities (*p* = 0.216).

Although not statistically significant, there was an observed trend for an increased prevalence of moderate–severe gingivitis with an increasing number of toothbrushes per day. There was no significant and linear association demonstrating an increase in maximum gingivitis with dental characteristics showing the same distribution pattern. The prevalence of moderate–severe gingivitis in women free of caries was stable (30.3% vs. 31.2%), while moderate–severe periodontitis was observed in 15% (30/207) of women. There was a significant difference between the mean BMI of women according to the severity of gingivitis. Thus, women with generalized gingivitis had a higher average BMI than those with localized gingivitis (*p* < 0.03).

### 3.5. Association between Gingivitis Cases (>10%) and Sociodemographic, Behavioral, and Clinical Variables

Table 4 presents the univariate and multivariate associations of sociodemographic, behavioral, and clinical variables with gingivitis as the dependent variable.

In univariate analysis, three variables, namely, age, education level, and fruit and vegetable consumption, were significantly associated with the risk of gingivitis. There was no significant difference in the risk of gingivitis between the very low, low, and moderate levels of education of the women, whereas the risk of gingivitis for the group with high education was 3.29 (95% CI [1.11–9.78]) times higher than that of the group of women with no education. In addition, the risk of gingivitis was associated with low fruit and vegetable consumption (<5 servings per day) (OR = 0.41; 95% CI [0.18–0.98]). The risk of gingivitis increased significantly with age (OR = 1.13; 95% CI [1.01–1.27]).

Oral health attitudes, such as brushing less than twice a day, did not result in statistically significant risk differences for gingivitis (*p* = 0.14). A higher risk of gingivitis was observed in association with increasing levels of dental biofilm. Unfavorable oral health status, such as CAL, was a high-risk factor for gingivitis; however, dental caries did not affect the risk of gingivitis in pregnant women.

In the multivariable model, physical activity and sedentary behavior were collinear, so only sedentary behavior was included because it was more strongly associated with the outcome. The association between gingivitis and fruit and vegetable consumption, dental biofilm, and CAL severity remained statistically significant after the inclusion of confounding variables. The crude (univariate effects) and adjusted (multivariate effects) ORs were similar, indicating that confounding effects are relatively small in this sample, at least for the variables studied. The risk of developing gingivitis decreased significantly with the consumption of more than five portions of fruits and vegetables per day (OR = 0.15; 95% CI [0.03–0.66]). At the same time, the risk of gingivitis increased with the percentage of dental biofilm (OR= 131.6; 95% CI [10.80–1619.71]) as well as the severity of CAL (OR = 7.70; 95% CI [3.16–18.92]). Similarly, the risk of gingivitis increased in women with professional activity (OR = 6.75; 95% CI [1.27–35.87]). An inverse association was observed with increasing BMI. The risk of gingivitis decreased with increasing BMI (OR= 0.76; 95% CI [0.63–0.93]).

## 4. Discussion

To the best of our knowledge, this is the first study initiated, with a strict definition of clinical gingival disease, an early stage of periodontal disease, to explore the clinical prevalence, severity, and risk factors of gingivitis cases in a 3-month pregnant population. Generally, the symptoms of gingivitis can be reversible and disappear because external or internal changes, such as the state of oral health, alteration of the immune system, oral hygiene practices, and diet, can disrupt the progression of the disease and interfere with the prevention and monitoring of the disease [40]. Gingivitis increases more quickly in pregnant women [17]. Gums become red and swollen due to inflammation that can be triggered or exacerbated by hormonal shifts [41]. Considerable differences in inflammatory biomarkers found in the oral biofilm were seen in pregnant women compared to nonpregnant women [9].

However, due to the lack of a clear definition, differences in methods of clinical assessment of gingival health, and the lack of a precise method for screening for gingivitis, it is currently difficult to compare the results of several epidemiological studies and infer a true difference in the prevalence of gingivitis [42]. The 2018 classification provided standardization for determining which patients with intact periodontium would be clinically diagnosed as having gingivitis in terms of prevalence and severity (EFP/AAP, 2018 [43]). Our research was based on this classification. To study the prevalence and severity of gingivitis cases, gingival bleeding was recorded on all teeth to define and grade gingivitis.

Gingival bleeding is the most easily identifiable clinical sign of gingivitis [44]. Approximately 88% of women in our study had clinical gingivitis. Bleeding was highly prevalent because 93% of pregnant women had at least one site with bleeding and 18.6% had 100% of sites with bleeding. Among the 220 women, 7% had no bleeding. Among Senegalese women at 3 months of pregnancy, 15% had moderate gingivitis, and 73% had severe gingivitis (30% or more sites with bleeding). Regarding the distribution of bleeding on each region of the dentition, each arch, and all surfaces, the exposed surfaces had different high bleeding scores. Our results are in accordance with the general findings of a previous clinical investigation among a sample of adolescents and/or young female subjects [45,46,47]. Generally, the gingiva of anterior teeth and their gingival papillae are more susceptible to the severity of gingivitis than that observed with the posterior teeth [48].

Little is known about the burden and risk factors for gingivitis among reproductive women at 3 months of pregnancy, not only in sub-Saharan Africa. Comparing our results on the prevalence of gingivitis with other studies should be considered with caution. These inconsistencies across these studies can be explained by several factors as follows: definitions of gingivitis, lack of a unified diagnostic standard, choice of indicators (Gingival index, Community Periodontal Index, Bleeding Index, etc. ), differences in the study design, low quality of the studies, date of investigation in some cases (>15 years), the unclear or inappropriate definition of cases (date of pregnancy) [11,49,50,51], and differences in age, ethnicity, access to dental care, and socioeconomic status of study populations. In the last 5 years, at the time of the new classification, studies on gingivitis cases in pregnant women are not numerous.

In this context, and with all the restrictions described, some former cross-sectional research showed that the percentage of pregnant women with gingivitis was 89% in Ghana, 86.2% in Thailand, and 47% in Brazil [16,52,53]. In a study published in 2016 including pregnant women less than 26 weeks’ gestation, 40% had gingival inflammation. Of those with gingival inflammation, 80% had localized gingival inflammation, and 20% had generalized gingival inflammation [54]. In a population of Kenyan women planning to conceive, Oyaro et al. found that 10% of the women had no gingivitis, 61% had mild gingivitis, 27% had moderate gingivitis, and 1% had severe gingivitis [50]. Therefore, our results can be considered in agreement with the trends of gingivitis prevalence described previously.

Socioeconomic factors such as maternal education (≥13 years) and professional activity were combined factors in the development of gingivitis in pregnant women at 3 months of pregnancy. In our study, the finding of a decreased frequency of gingivitis in women with the lowest education level vs. the highest education level was not consistent with the findings of other studies [11,12,55,56], where a higher level of education was associated with better oral health. We have no rational explanation to produce other than to express a reserve on the low power of the statistical analysis of our study related to the high number of classes and consequently to the low intraclass numbers. However, the impact of the use of traditional toothbrushes made of fibers rich in organic substances more commonly known as “sothiou” or “siwak”, which are used predominantly in the lower classes or in conjunction with a regular toothbrush, on gingival health, which was not taken into consideration in our study, should not be neglected [57]. Interestingly, Oyaro et al. recently reported in a pregnant Kenyan population that in univariable analyses, lower moderate–severe gingivitis was observed in association with increasing levels of education and monthly household income. In a multivariable analysis including location, education, and income, the association with the site was similar, while the associations with education and income were attenuated and no longer statistically significant [50].

In addition, somewhat disconcertingly, there was no difference in gingival condition between women who brushed twice a day and those who brushed less frequently. While applying the same logic to the positive effect of traditional toothbrush use, we should keep in mind that self-reported dental hygiene may not reflect actual dental hygiene, and if participants report brushing twice a day, they may do so much less frequently.

Clinical variables such as PI and CAL were positively associated with gingivitis, while the observed dental health of caries-free patients did not impact the gingival condition. Obesity was positively associated with gingivitis, although many common risk factors of periodontal diseases, such as smoking, alcohol use, and other chronic diseases, were low or nonexistent in this population. Additionally, older pregnant women could have the highest association with gingival disease, as described in the univariate analysis.

Both lifestyle and dental health are weighted differently for people depending on their culture, socioeconomic status, and level of education [58]. Therefore, cultural and economic factors were studied as affecting oral health outcomes and were considered in characterizing women in several studies [59]. However, in our study, there were no effects on the results.

The association observed in bivariate analysis between BMI and gingival conditions can be explained by the fact that the adipose tissue of patients with excessive weight secretes inflammatory mediators, such as tumor necrosis factor-alpha, interleukin 6, and C-reactive protein, which can make the host more susceptible to inflammation [9,60,61]. Therefore, overweight or obese patients may have higher levels of periodontal inflammation and destruction, even in the presence of normal amounts of bacterial biofilm, compared to normal-weight patients [62].

Most study participants reported brushing their teeth twice a day. The gingival status of the pregnant women included in this survey was unsatisfactory, and the majority had BOIB. This inconsistency between gingival condition and reported dental hygiene practice may be a result of the women having not acquired adequate brushing techniques [63,64,65]. This observation is not limited to the study population. For example, comparable results have been reported for dental students, whose bleeding rate was 84%. Only an effective and efficient brushing technique combined with interdental brushing allows for optimal disruption of interdental biofilm. In particular, it has been found that using interdental cleaning in combination with tooth brushing can reduce gingivitis or dental biofilm more than tooth brushing alone [37,66]. During pregnancy or puberty, estrogen and progesterone levels increase, leading to an increase in gingival vascularization and inflammation that must also be taken into account [67]. Female sex steroid hormones may thus indirectly contribute to gingival and periodontal diseases by exacerbating the response of periodontal tissue to dental biofilm [68]. Evidence-based recommendations for concrete procedures and practices to significantly enhance the gingival health of pregnant women and decrease the risk of periodontal disease and preterm birth or preeclampsia were not common [13]. The best option for periodontal tissue health is better oral hygiene [46]. However, in practice, the question is how to proceed and what strategies to adopt. Given the multifactorial nature of inflammation in pregnant women, occupational prophylaxis is necessary [69]. The effectiveness of brushing alone cannot be guaranteed in this high-risk period for gingival health. Currently, the therapy of choice is the mechanical removal of dental plaque above and below the gum line [70]. The challenge is to organize geographical and financial accessibility to intensive health monitoring and oral prophylaxis of these pregnant women in low-income countries, where access to oral health care for the population is estimated at 35% [71].

The bacterial biofilm that forms a dental plaque is an important factor in triggering gingivitis [4,72,73]. In our study, the odds of gingival disease were significantly increased in women with the highest PD. If the dental biofilm indicates inadequate oral hygiene, plaque accumulates more rapidly around inflamed gums than around gums without inflammation. The biofilm reacts immunologically with the host to produce an inflammatory infiltrate in the gingival tissue, leading to the development of gingivitis [74]. The extent of gingivitis increases as the amount and location of plaque buildup increase, further confirming the association of large amounts of plaque with generalized gingivitis [33].

Participants had a minimal recession, and the loss of attachment estimates was driven primarily by probing depth. This may be a result of the young age distribution of the population. However, our study found positive results with respect to CAL during pregnancy [75,76]. Pregnant women remain at increased risk of recurrent progression of periodontitis. On a reduced periodontium, clinical gingival health was characterized by an absence of bleeding on probing, erythema, edema, and patient symptoms in the presence of reduced clinical attachment and bone levels [43]. In nonperiodontitis patients, there was no current evidence for an increased risk of periodontitis on a reduced periodontium. However, evidence has demonstrated that a patient may achieve periodontal stability. Periodontal stability is characterized by successful treatment through control of local and systemic risk factors, resulting in minimal (<10% of sites) bleeding, no probing depths of 4 mm or greater than bleed on probing, optimal improvement in other clinical parameters, and lack of progressive periodontal destruction [77]. Although most inflammatory changes, such as gingival changes, gingivitis, and sometimes localized growth of gingival tissues, will disappear within a few months after delivery, previous epidemiological evidence has suggested that females during pregnancy are more likely to experience periodontal disease caused by a variety of factors [18]. However, gingivitis in pregnant women very rarely turns into periodontitis, as shown by the loss of connective tissue attachment [2]. Indeed, progesterone has a suppressive action on gingival fibroblasts, inhibiting the production of matrix metalloproteinases, the enzyme responsible for the destruction of collagen fibers [78,79].

Healthy lifestyle habits such as eating fruits and vegetables are considered part of an overall behavior impacting oral health [80]. In our study, food intake was associated with gingival bleeding cases. Our data suggested that increased portions of fruits and legumes (≥5 portions per day) were associated with a decreased incidence of gingivitis in pregnant patients. Good nutritional patterns are protective against periodontitis [81]. Liu et al. reported that subjects with bad diets had an odds ratio of 4.22 compared to subjects with excellent diets, indicating poor dental health after adjustment for confounding factors [82]. Poor eating habits may be an important risk factor for oral inflammation and gingivitis [83]. Consumption of less than recommended portions of fruits and vegetables was associated with higher odds of bleeding. In addition, fewer portions of healthy foods mean fewer nutrients that protect gum tissue and support the immune system [84,85]. Reducing oxidative stress and increasing antioxidant intake through certain nutritional components benefit gingival and periodontal inflammation due to modification of the host immune response [86].

Conceptually, our results contribute to understanding the determinants of gingival bleeding in pregnant women. They support the clinical definitions and hypotheses of the 2018 classification of gingivitis, which identifies three broad categories of gingival disease: plaque-induced gingivitis, mediated by systemic or local risk factors, and nondental plaque biofilm-induced gingival disease due to higher levels of estrogen and progesterone [43]. Evaluating bleeding using a specific method while citing EFP/AAP 2018 is not inconsistent, as the “clinical” endpoint of the classification is bleeding vs. no bleeding with a cut-off of 10% of the sites listed per individual.

A strength of our study is the control for potential confounders known to affect dietary intakes and oral health practices, such as age, tooth brushing, and education. In contrast, other lifestyle factors, such as alcohol consumption and smoking, were not assessed because of societal and cultural norms that would limit the involvement and reporting of these factors. Clinical procedures and assessments were standardized for this investigation, and a clinical examiner calibrated with greater than 90% reliability for each clinical index performed all examinations under constant lighting conditions.

Our research was limited to identifying the severity of the extension of gingival inflammation cases and not the severity of the inflammatory condition. The definition and classification of a site-level gingival inflammatory condition (i.e., a “gingivitis site”) are separate from the definition and classification of a gingivitis case (i.e., a patient with gingivitis), and a “gingivitis site” is not necessarily equivalent to a GC. Although it may be useful for investigational research purposes, the use of quantification indices to systematically grade BOP at site levels may lead to variability in the grading scale that is challenging to define during periodontal examinations [33].

Our study conclusions are, however, limited by its cross-sectional exploratory design, which can only provide evidence of association. Proving causality requires longitudinal studies that follow pregnant women from the early stages of pregnancy to its end so that temporality can be ascertained. A study with a larger sample size and randomly selected subjects is also needed so that conclusions can be more confidently generalized to the target population. Finally, we recall the potential bias in the results related to the association of the variables such as level of education, number of daily brushings, and severity of the extension of gingivitis related to the nonconsideration of the use of traditional toothbrushes.

## 5. Conclusions

Our analyses suggested that gingivitis cases were high among Senegalese women at 3 months of pregnancy. A total of 88% had clinical gingivitis. Fifteen percent had localized gingivitis, and seventy-three percent had generalized gingivitis. Bleeding was also highly prevalent. The age of women, plaque index, clinical attachment level, BMI, and fruit and vegetable consumption were identified as risk factors for gingivitis. Future studies should assess the interaction between gingivitis from biofilm and gingivitis from systemic disorders to highlight the importance of enabling pregnant women to manage their oral health by adopting healthy practices.

## Figures and Tables

**Table 1:** Demographic characteristics and lifestyle habits of pregnant women.

Characteristics	Overall (N = 220)
**Age (years)**	
Mean ± SD	23.6 ± 4.6
Median [IQR]	23 [20.0–26.0]
**Pregnancy age (weeks)**	
Mean ± SD	12.3 ±0.7
Median [IQR]	12 [12.0–13.0]
**Professional activity, n/N (%)**	
No	139/220 (63.2)
Yes	81/220 (36.8)
**Residence, n/N (%)**	
Urban	209/220 (95.0%)
Rural	11/220 (5.0%)
**BMI (kg/m^2^),**	
Mean ± SD	23.1 ± 4.5
Median [IQR]	22.6 [22.2–25.1]
**Education, n/N (%)**	
No	47/220 (21.4%)
1–6 years	34/220 (15.5%)
7–12 years	59/220 (27.0%)
≥13 years	79/220 (36.1%)
**Fruits and vegetables consumption, n/N (%)**	
<5 portions by day	161/220 (74.5)
≥5 portions by day	55/220 (25.5)
**Physical activity, n/N (%)**	
No <1500 metabolic equivalent of task/week	55/220 (24.8)
Yes ≥1500 metabolic equivalent of task/week	165/220 (75.2)
**Sedentary behavior, n/N (%)**	
Active	11/220 (5.0)
Sedentary	209/220 (95)
**Toothbrushing frequency, n/N (%)**	
1/day	66/220 (30.0)
≥2/day	154/220 (70.0)

**Table 2:** Clinical parameters of pregnant women.

Characteristics	Overall (N = 220)
**Caries free, n/N (%)**	
Yes	68/220 (68.9)
No	151/220 (31.1)
**Decayed teeth**	
Mean ± SD	1.8 ± 2.5
Median [IQR]	1.0 [0.0–3.0]
**Missing teeth**	
Mean ± SD	0.7 ± 1.3
Median [IQR]	0.0 [0.0–1.0]
**Filled teeth**	
Mean ± SD	0.1 ± 0.5
Median [IQR]	0.0 [0.0–0.0]
**DMFT (Decayed, Missing, Filled teeth)**	
Mean ± SD	2.7 ± 3.1
Median [IQR]	2.0 [0.0–4.0]
**Plaque index**	
Mean ± SD	0.5 ± 0.5
Median [IQR]	0.4 [0.1–0.8]
**Gingival index**	
Mean ± SD	0.2 ± 0.4
Median [IQR]	0.1 [0.0–0.3]
**Pocket depth (mm)**	
Mean ± SD	2.2 ± 0.6
Median [IQR]	2.2 [1.8–2.6]
**Clinical attachment level (mm)**	
Mean ± SD	2.0 ± 1.1
Median [IQR]	2.3 [1.4–2.7]
**Bleeding on interdental brushing (%)**	
Mean ± SD	58.5 ± 34.0
Median [IQR]	66.7 [28.8–92.1]

**Table 3:** Distribution of gingivitis extension cases according to sociodemographic, behavioral, and clinical variables.

Characteristics	No/Mild N = 26	Localized Gingivitis N = 33	Generalized Gingivitis N = 161	*p* Value
**Age** (years)	21.8 (±3.7)	24.0 (±4.0)	23.8 (±5.0)	0.060 ^a^
**Pregnancy age** (weeks)	12.4 ± 0.7	12.3 ± 0.8	12.3 ± 0.7	0.516 ^a^
**Professional activity**, n/N (%)				
No	7 (26.9)	16 (48.5)	103 (64.0)	0.216 ^b^
Yes	19 (73.1)	17 (51.5)	58 (36.0)	
**Residence, n/N (%)**				0.457 ^b^
Urban	26 (100)	31 (93.9)	152 (94.4)	
Rural	-	2 (6.1)	9 (5.6)	
**BMI** (kg/m^2^)	23.4 ± 4.3	21.2 ± 3.3	23.4 ± 4.6	0.030 ^a^
**Education**, n/N (%)				0.246 ^b^
No	10 (38.5)	5 (15.2)	33 (20.0)	
1–6 years	3 (11.5)	8 (24.2)	23 (14.4)	
7–12 years	7 (26.9)	9 (27.3)	43 (26.9)	
≥13 years	6 (23.1)	11 (33.3)	62 (38.8)	
**Fruits and vegetables consumption**				0.108 ^b^
<5 portions/day	15 (57.7)	26 (78.1)	122 (76.6)	
≥5 portions/day	11 (42.3)	7 (21.9)	37 (23.4)	
**Physical activity**				0.699 ^b^
Yes	8 (30.8)	7 (21.2)	40 (24.8)	
No	18 (69.2)	26 (78.8)	121 (75.2)	
**Sedentary behavior**				0.133 ^b^
Active	3 (11.5)	-	8 (5.0)	
Sedentary	23 (88.5)	33 (100.0)	151 (95.0)	
**Toothbrushing frequency**				0.333 ^b^
1/day	11 (42.3)	10 (30.3)	45 (28.0)	
≥2/day	15 (57.7)	23 (69.7)	116 (72.0)	
**Caries free**				0.994 ^b^
Yes	8 (30.8)	10 (30.3)	50 (31.2)	
No	18 (69.2)	23 (69.7)	110 (68.8)	
**Plaque index**	1.44 ± 0.50	1.89 ± 0.59	2.35 ± 0.48	<0.001 ^a^
**CAL (mm)**	0.52 ± 0.94	1.27 ± 1.33	2.33 ± 0.85	<0.001 ^a^

^a^ Kruskal–Wallis rank sum test; ^b^ Pearson’s chi-squared test.

**Table 4:** Univariate and multivariate logistic regression models of the association between gingivitis and sociodemographic, behavioral, and clinical variables.

Characteristic	Healthy (n = 26)	Gingivitis (n = 194)	Unadjusted OR [95% CI]	*p* Value	Adjusted OR [95% CI]	*p* Value
**Age** (years)	21.8 ± 3.7	21.1 ± 3.1	1.13 [1.01–1.27]	0.03	1.14 [0.98–1.32]	0.09
**Pregnancy age** (weeks)	12.4 ± 0.7	12.3 ± 0.7	0.76 [0.45–1.3]	0.32		
**Professional activity**, n/N (%)						
No	7 (26.9)	119 (61.3)	1			
Yes	19 (73.1)	95 (38.7)	1.68 [0.67–4.18]	0.27	6.75 [1.27–35.87]	0.02
**Residence, n/N (%)**						
Urban	26 (100)	183 (94.3)	1			
Rural	-	11 (5.7)	6016402.22 [0.00-Inf]	0.99		
**BMI** (kg/m^2^)	23.9 ± 4.7	22.9 ± 4.7	0.98 [0.90–1.08]	0.90	0.76 [0.63–0.93]	0.01
**Education**, n/N (%)						
No	10 (38.5)	38 (19.1)	1			
1–6 years	3 (11.5)	31 (15.5)	2.80 [0.70–11.02]	0.14		
7–12 years	7 (26.9)	52 (26.8)	2.01 [0.70–5.75]	0.19		
≥13 years	6 (23.1)	73 (37.6)	3.29 [01.11–9.78]	0.03		
**Fruits and vegetables consumption**						
<5 portions/day	15 (57.7)	148 (76.3)	1			
≥5 portions/day	11 (42.3)	46 (23.7)	0.41 [0.18–0.98]	0.04	0.15 [0.03–0.66]	0.01
**Physical activity**						
Yes	18 (69.2)	147 (75.7)	1			
No	8 (30.8)	47 (24.3)	1.39 [0.57–3.39]	0.47		
**Sedentary behavior**						
Active	3 (11.5)	10 (5.1)	1			
Sedentary	23 (88.5)	184 (94.9)	3.0 [0.74–12.06]	0.12	0.53 [0.05–5.81]	0.92
**Toothbrushing frequency**						
1/day	11 (42.3)	55 (28.4)	1			
≥2/day	15 (57.7)	139 (71.6)	1.86 [0.80–4.31]	0.14	0.90 [0.26–3.10]	0.82
**Caries free**						
Yes	18 (69.2)	134 (69.1)	1			
No	8 (30.8)	60 (30.9)	1.01 [0.42–2.46]	0.97	0.95 [0.25–3.60]	0.94
**Plaque index**	0.19 ± 0.25	0.59 ± 0.48	27.66 [4.81–159.17]	<0.001	131.6 [10.80–1619.71]	<0.001
**CAL** (mm)	0.52 ± 0.94	1.95 ± 1.06	3.71 [2.34–5.87]	<0.001	7.7 [3.16–18.92]	<0.001

## Data Availability

The data presented in this study are available on request from the corresponding author.

## Supplementary Materials

The following supporting information can be downloaded at https://www.mdpi.com/article/10.3390/jcm12093349/s1, Table S1: STROBE checklist.
